# Motion Control System for USV Target Point Convergence

**DOI:** 10.3390/s24206589

**Published:** 2024-10-12

**Authors:** Jian Zhou, Hui Zhang, Kai Liu, Linhan Ma, Yanxia Yang, Zhanchao Fan

**Affiliations:** School of Information and Automation Engineering, Qilu University of Technology, Shandong Academy of Sciences, Jinan 250300, China; 18853104864@163.com (J.Z.); liukaiyy2017@163.com (K.L.); mlinhan906@163.com (L.M.); yangyanxia0711@163.com (Y.Y.); bhrqne5i5j@163.com (Z.F.)

**Keywords:** unmanned surface vehicles, point-to-point tracking, heading control, path following, dynamic positioning

## Abstract

The goal of this paper is to establish a motion control system for unmanned surface vehicles (USVs) that enables point-to-point tracking and dynamic positioning. This includes the heading control and path following control of USVs. A hardware and software platform for USVs using microcontrollers is designed. This paper presents the construction of a kinematics and dynamics model for an unmanned catamaran. The motion process is divided into two segments. In the target point tracking segment, the heading coordinate system and the ship coordinate system are established. Based on these, a control method using differential steering to track the desired yaw angle is designed to improve the tracking efficiency. And the accuracy of heading keeping and path following is improved by combining the cascade PID controller. In the dynamic positioning segment, a self-adjusting mechanism is designed, thereby enhancing the flexibility of thrust distribution and improving the accuracy of the USV’s positioning retention in wind and wave environments. Finally, experimental validation is carried out to verify the effectiveness of the design proposed in this paper by issuing control commands and saving the return data through the upper computer, and then analyzing the return data with MATLAB (R2022b, MathWorks, Natick, MA, USA).

## 1. Introduction

In recent years, the application of USVs in ocean space has received increasing attention [1,2,3]. A USV is an intelligent, multifunctional surface platform controlled by remote control or autonomous navigation [4]. It plays an important role in the fields of marine resources exploration, maritime patrol, and meteorological observation. In order to enhance the sustainable development of marine resources through the utilization of USVs, research on the control of USVs is particularly important [5]. Currently, the motion research of USVs includes heading control [6], path following control [7], and trajectory tracking control [8], among which, the correlation between heading control and path following is more widely studied.

The heading control of a USV is designed to rapidly change the actual yaw angle to the reference yaw angle and to minimize the amount of overshoot. In previous research, PID controllers were first proposed and applied to real ship heading control [9]. However, in simulations and field experiments, the USV is susceptible to model perturbations and environmental disturbing forces, making it difficult for the PID controller to maintain a consistent control performance, and parameters need to be readjusted to stabilize the system [10,11]. Therefore, a control method with good robustness and adaptability is needed. Wu et al. [12] added a tracking differentiator to a model-free adaptive controller and designed a controller for complex systems such as nonlinear, time-delayed, time-varying, and strongly coupled. Bu et al. [13] considered the data-driven control (DDC) problem for a class of non-affine nonlinear systems with output saturation based on model-free adaptive control theory. In 2018, Li et al. [14] designed a PID-MFA cascade controller based on model-free adaptive control theory to realize the heading control of USVs through angular velocity guidance. In 2019, to address the problem that the USV heading subsystem does not satisfy the quasi-linear assumption, Liao et al. [15] redefined the output of the USV as a linear sum of heading and angular velocity, and based on this redefinition proposed a free adaptive control method for the compact format model. However, the above methods are more dependent on the accuracy of the motion model, complicated in design, have high environmental uncertainty, and have not been applied in real environments. Based on the heading coordinate system and ship coordinate system, this paper designs a method of tracking and maintaining the desired heading yaw using differential speed control, and establishes a cascade PID controller to optimize the control accuracy. Finally, it is practically applied to USVs and achieves a good heading control effect.

Path following is one of the fundamental motion control problems in the autonomous control of USVs. Path following is defined as a USV following a time-independent path at a certain speed [16]. Currently, the well-known bootstrap method is LOS (Line of Sight) bootstrap [17]. To address external marine environmental disturbances such as constant or time-varying winds, waves, and currents, Fossen et al. proposed ILOS (Integral LOS) and ALOS (Adaptive LOS) based on proportional LOS guidance methods [18]. As the core of the path-following control system, the control algorithm is the key to determining whether the USV can accomplish the intended task.

A system that maintains a surface vehicle in a predetermined position and heading by means of an appropriate propeller and propeller action is known as a dynamic positioning (DP) system. The technology is applicable to ships, which can use their own propulsion instead of traditional mooring systems to maintain the desired position and heading in the event of disturbances on the water [19], and the problem of controlling the dynamic positioning system of ships has been a hot topic of research and discussion among international experts and scholars [20]. Currently, DP has developed into an important technology for deep-sea resource exploration and is widely used on drill ships, cable-laying vessels, supply vessels, survey vessels, and offshore platforms [21]. This emphasizes the considerable theoretical importance and practical utility of DP [22]. However, the dynamic positioning system is usually applied to large ships, while the application in small USVs is less common. One of the research objectives of this paper is to design a simple and efficient dynamic positioning device that can be practically applied to small USVs, which can help small USVs keep in the predetermined position for operation and reduce the interference of wind and waves on the vehicles.

The rest of the paper is as follows. Section 2 designs the general structure of the USV. Section 3 models the dual-propeller differential speed USV to provide theoretical support for the later scheme. Section 4 proposes a solution for heading tracking and keeping, and Section 5 describes the working principle of the adaptive adjustment mechanism and carries out real ship experiments to verify the above solution. Finally, the paper is summarized in Section 6.

## 2. Overall Design of the USV

### 2.1. Hull Structure

The overall design of the USV is the prerequisite and foundation for researching the design of autonomous navigation control for USVs. The hull structure of the USV employed in this paper is a dual-propeller catamaran structure. The control system of the USV is mainly comprised of two parts: the ship-based system and the shore-based system.

**Figure 1:**
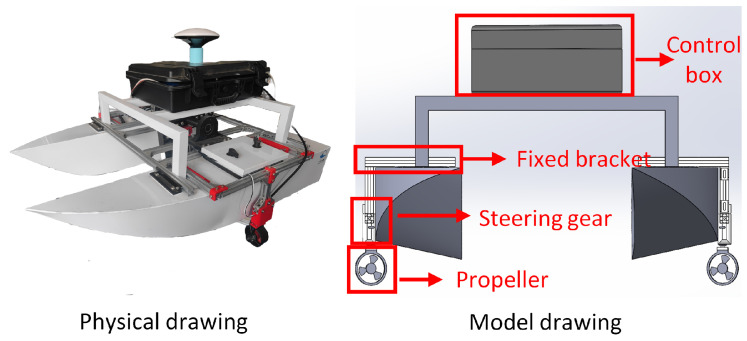
Physical and model drawing of hull structure.

As shown in Figure 1, this paper enhances the traditional catamaran structure. In the traditional catamaran, the propeller is typically fixed at the rear of the hull. The disadvantage of this structure is that it can only exert longitudinal force on the hull. To overcome this problem, this paper employs fixed brackets to connect the propeller to waterproof steering gears and positions them on both sides of the USV. The steering gear rotates to drive the propellers to rotate, enabling the thrust generated by the propellers to be distributed in all directions. The ship-based system is located in the control box.

### 2.2. Overall Design of Ship-Based System

**Figure 2:**
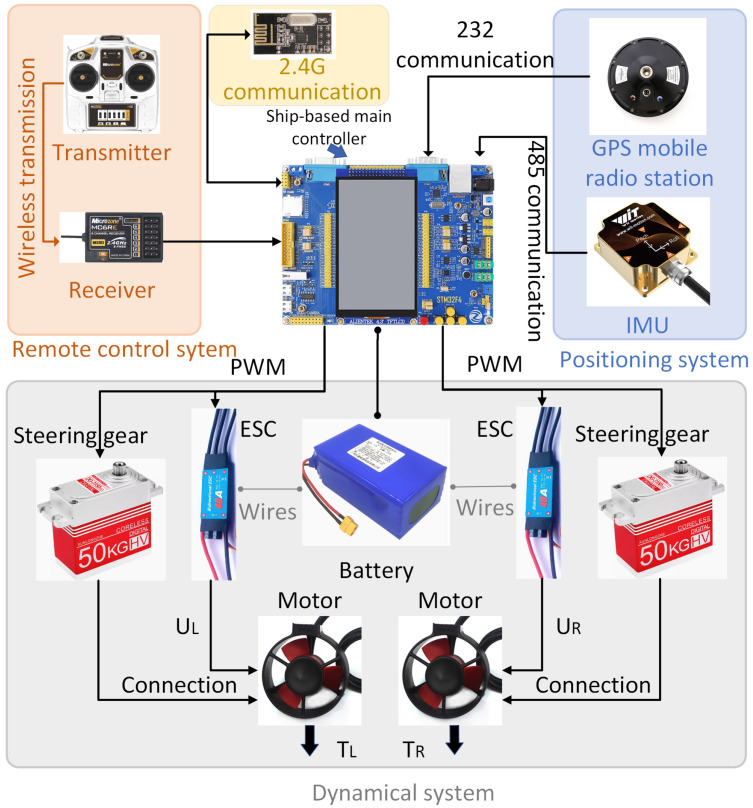
Design of ship-based control system.

As shown in Figure 2, the ship-based control system is composed of four main components, namely ship-based main controller, positioning system, remote control system, and propulsion system.

The design idea of the positioning system is to utilize the **Global Positioning System** (GPS) mobile station to acquire the latitude, longitude, real-time speed, and other information of the USV. Subsequently, through the Inertial Measurement Unit (IMU), the yaw angle and steering angular velocity of the USV’s bow, among other information, are obtained. The aforementioned information is transmitted to the ship-based main controller via 232 communication and 485 communication, respectively. After analysis by the main controller, the position and attitude information of the USV can be obtained.

The design of the remote control system is to use the transmitter to output control instructions. According to the control instructions, the transmitter sends data to the receiver. Then, through the SBUS to serial module, the data are sent to the ship-based main controller. The main controller parses the data and finally obtains the control code to control the propellers.

The design idea of the propulsion system is to utilize electronic speed controls to control the brushless motors to drive the movement of the USV. In the target point tracking segment, the dual-propellers USV employs the rotational speed difference to turn, which significantly reduces the turning radius and enhances flexibility. Due to the real-time acquisition and processing of information, when the desired yaw angle information is received, the main controller outputs PWM waves to control the propellers in real time. Moreover, the dual-propellers USV saves the time by steering through non-essential paths and is capable of adjusting to the desired yaw angle more quickly, thus shortening the response time. In the dynamic positioning segment, the position of the USV is corrected through the cooperation of the steering gears and propellers. The system is powered by lithium battery packs.

### 2.3. Overall Design of Shore-Based System

**Figure 3:**
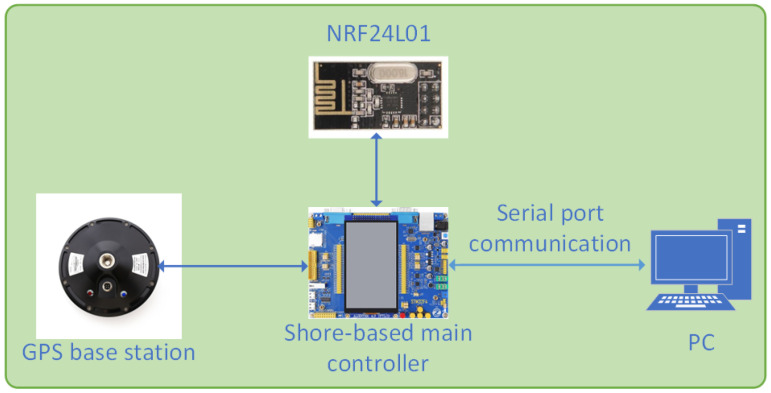
Design of shore-based control system.

As shown in Figure 3, the design idea of the shore-based system is that the main controller of the shore-based system receives latitude, longitude, and target point parameters information set by the upper computer through serial communication. Then, it sends this information to the ship-based main controller via wireless communication. The ship-based control system receives the information and controls the USV to navigate autonomously. Simultaneously, real-time latitude, real-time longitude, real-time yaw angle, and other information are also transmitted back to the main controller of the shore base via wireless communication, Subsequently, the information is visualized and stored through the upper computer. The GPS mobile station utilizes the differential data sent by the GPS base station to perform RTK accurate positioning.

## 3. Mathematical Modeling of USV

### 3.1. Establishment of Coordinate Systems

The motion of the USV is classified as a six-degree-of-freedom state of motion, which is generally described based on two coordinate systems: a North-East-Down coordinate system, with the center of the Earth as the origin of the coordinates, and a body-fixed coordinate system, with the USV itself as the origin of the coordinates.

**Figure 4:**
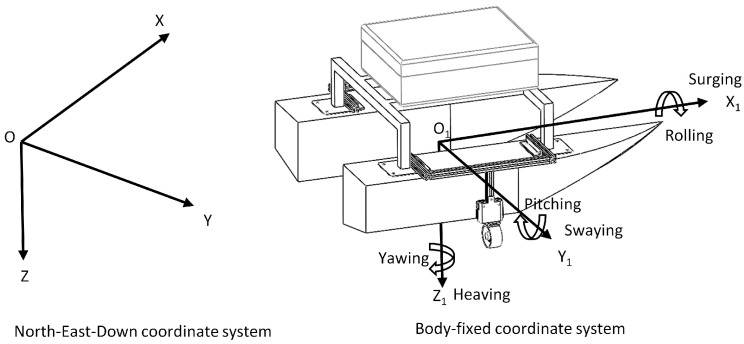
Schematic diagram of the coordinate system.

As shown in Figure 4, in the North-East-Down coordinate system OXYZ, the *X*-axis points north, the *Y*-axis points due east, and the *Z*-axis points to the center of the earth. The coordinate system $O_{1}X_{1}Y_{1}Z_{1}$ refers to the body-fixed coordinate system, with the $X_{1}$-axis pointing in the direction of the ship’s bow, the $Y_{1}$-axis perpendicular to the $X_{1}$-axis pointing to the right side of the ship, and the $Z_{1}$-axis perpendicular to the $X_{1}O_{1}Y_{1}$ plane pointing to the center of the earth. The physical quantities of the body-fixed coordinate system are shown in Table 1.

### 3.2. Kinematic Model

We generally ignore the effects of heave, roll, and pitch motions. The six-degree-of-freedom motion is simplified to a three-degree-of-freedom motion of surge, sway, and yaw. From this, combining Equations (1) and (2) can be simplified to obtain a simplified mathematical model:

(1) $$\begin{array}{c} \left[\begin{array}{c} \dot{x}\\ \dot{y}\\ \dot{z} \end{array}\right]=\left[\begin{array}{ccc} cos\theta cos\psi & sin\phi sin\theta cos\psi -cos\phi sin\psi & cos\phi sin\theta cos\psi +sin\phi sin\psi \\ cos\theta sin\psi & sin\phi sin\theta sin\psi +cos\phi cos\psi & cos\phi sin\theta sin\psi -sin\phi sin\psi \\ -\sin\theta & sin\phi cos\theta & cos\phi cos\theta \end{array}\right]\left[\begin{array}{c} u\\ \nu \\ w \end{array}\right] \end{array}$$

(2) $$\left[\begin{array}{c} \phi \\ \dot{\theta }\\ \dot{\psi } \end{array}\right]=\left[\begin{array}{ccc} 1 & sin\phi tan\theta & cos\phi tan\theta \\ 0 & cos\phi & -sin\phi \\ 0 & sin\phi sec\theta & cos\phi sec\theta \end{array}\right]\left[\begin{array}{c} p\\ q\\ r \end{array}\right]$$

(3) $$\left\{\begin{array}{c} \dot{x}=u\cos\psi -\nu \sin\psi \\ \dot{y}=u\sin\psi +\nu \cos\psi \\ \dot{\psi }=r \end{array}\right.$$

### 3.3. Dynamics Model

The position and attitude angle changes of a USV under the influence of external forces and moments are illustrated by means of a dynamics model. To facilitate our analysis of the model, the following assumptions are made:

(1) Since the USV is moving in the horizontal plane, ignoring the effects of surge, sway, and yaw motions, z=0,w=0,ϕ=0,p=0,θ=0,q=0.
(2) The mass of the hull is evenly distributed and symmetrical, so the values of $I_{xy}$ and $I_{yz}$ are both 0.
(3) The origin of the body-fixed coordinate system is set at the center of gravity of the USV so that the center of gravity coordinates $x_{1g}$, $y_{1g}$, and $z_{1g}$ are all 0.

Based on the above assumptions, a mathematical model of the horizontal motion of the USV can be constructed.

(4) $$M\dot{\nu }+C\left(\nu \right)\nu +D\left(\nu \right)\nu =\tau +\tau_{E}$$

In Equation (4), $v={\left[u,v,r\right]}^{T},\;\tau ={\left[\tau_{u},\;\tau_{v},\;\tau_{r}\right]}^{T}$, and $\tau_{u}$, $\tau_{v}$, and $\tau_{r}$ are the ship’s longitudinal thrust, transverse thrust and turning moment, respectively. In particular, it is noted that in the target point tracking segment $\tau_{v}$ = 0 at this time, since the thrust only acts in the longitudinal direction of the USV. In the dynamic positioning segment, the USV is no longer steered by differential speed, so at this point $\tau_{r}$ = 0, $\tau_{E}={\left\lceil \tau_{E,u},\tau_{E,v},\tau_{E,r}\right\rceil }^{T}$ denotes the external environmental perturbation. The specific expressions of each matrix and the meanings they represent are explained below:

(1) Inertia matrix

(5) $$M=\left[\begin{array}{ccc} m_{11} & 0 & 0\\ 0 & m_{22} & m_{23}\\ 0 & m_{23} & m_{33} \end{array}\right]$$

*M* represents the inertia matrix of the system, which is a positive definite matrix, $m_{11}=m-X_{\dot{u}},m_{22}=m-Y_{\dot{v}},m_{33}=I_{z}-N_{\dot{r}}$. *m* denotes mass, $X_{\dot{u}},Y_{\dot{v}}$, and $N_{\dot{r}}$ denote longitudinal, transverse, and navigational additional mass and moment coefficients of inertia, and $I_{z}$ denotes moment of inertia.

(2) Coriolis matrix

(6) $$C\left(\nu \right)=\left[\begin{array}{ccc} 0 & 0 & -m_{22}\nu -m_{23}r\\ 0 & 0 & m_{11}u\\ m_{22}\nu +m_{23}r & -m_{11}u & 0 \end{array}\right]$$

C(v) reflects the effects that a ship undergoes during a particular rotational motion at sea, taking into account the role of additional mass. Although this matrix does not directly represent the actual forces and moments exerted on the ship, its influence is critical in the study of ship motion in a non-inertial reference system.

(3) Hydrodynamic damping coefficient matrix

(7) $$D\left(\nu \right)=\left[\begin{array}{ccc} d_{11} & 0 & 0\\ 0 & d_{22} & d_{23}\\ 0 & d_{32} & d_{33} \end{array}\right]$$

Under ideal fluid conditions, D(v) exhibits asymmetric and strictly positive definite properties. $d_{11}=-X_{u}$, $d_{22}=-Y_{v}$, $d_{33}=-N_{r}$. $X_{u},Y_{v}$, and $N_{r}$ are linear damping coefficients.

Most ships have left–right symmetry and move in a two-dimensional plane. In the ideal situation, the USV is assumed to be a rigid body so that the interaction between each mass element is neglected. To simplify the model and consider the properties of the inertia matrix, *M*, and the damping matrix, *D*, at low speeds, Fossen [23] noted that the non-diagonal elements were insignificant compared with the diagonal elements, and therefore ignored the influence of these non-diagonal elements.

(8) $$M=\left[\begin{array}{ccc} m_{11} & 0 & 0\\ 0 & m_{22} & 0\\ 0 & 0 & m_{33} \end{array}\right]$$

(9) $$C\left(\nu \right)=\left[\begin{array}{ccc} 0 & 0 & -m_{22}\nu \\ 0 & 0 & m_{11}u\\ m_{22}\nu & -m_{11}u & 0 \end{array}\right]$$

(10) $$D\left(\nu \right)=\left[\begin{array}{ccc} d_{11} & 0 & 0\\ 0 & d_{22} & 0\\ 0 & 0 & d_{33} \end{array}\right]$$

The simplified equations for the kinematics and dynamics of the USV can be expressed as:

(11) $$\left\{\begin{array}{c} \dot{x}=u\cos\psi -\nu \sin\psi \\ \dot{y}=u\sin\psi +\nu \cos\psi \\ \dot{\psi }=r\\ \dot{u}=\frac{m_{22}}{m_{11}}vr-\frac{d_{11}}{m_{11}}u+\frac{\tau_{u}+\tau_{E,u}}{m_{11}}\\ \dot{v}=-\frac{m_{11}}{m_{22}}ur-\frac{d_{22}}{m_{22}}v+\frac{\tau_{v}+\tau_{E,v}}{m_{11}}\\ \dot{r}=\frac{m_{11}-m_{22}}{m_{33}}u\nu -\frac{d_{33}}{m_{33}}r+\frac{\tau_{r}+\tau_{E,r}}{m_{33}} \end{array}\right.$$

Considering that the catamaran, which has a uniform mass distribution, is chosen as the main body of the USV in this paper, we simplify the hull as a mass point in performing the longitudinal force analysis. In this case, the combined force of the propulsive forces in the longitudinal and transverse directions are expressed as:

(12) $$\tau_{u}=\left(F_{l}+F_{r}\right)\cdot \sin\varphi$$

(13) $$\tau_{v}=\left(F_{l}+F_{r}\right)\cdot \cos\varphi$$

$F_{l}$ represents the effective thrust of the left propeller and $F_{r}$ represents the effective thrust of the right propeller. φ is the angle between the line where the combined force of the propellers is located and the line where $O_{1}Y_{1}$ is located in the body-fixed coordinate system. The value of φ is in the range [0, 90].

In the target point tracking segment, the propellers’ thrust always acts on the longitudinal direction of the catamaran, so $\varphi =90,\tau_{u}=F_{l}+F_{r},\tau_{v}=0.$ In this case, the expression for the moment produced by the propellers is:

(14) $$\tau_{r}=\left(F_{l}-F_{r}\right)\cdot B_{d}$$

$B_{d}$ represents the distance of the two propellers relative to the center axis of the hull.

In the target point tracking segment, we can further reformulate the dynamics equation of the USV as:

(15) $$\left\{\begin{array}{c} \dot{x}=u\cos\psi -v\sin\psi \\ \dot{y}=u\sin\psi +v\cos\psi \\ \dot{\psi }=r\\ \dot{u}=\frac{m_{22}}{m_{11}}vr-\frac{d_{11}}{m_{11}}u+\frac{F_{l}+F_{r}+\tau_{E,u}}{m_{11}}\\ \dot{v}=-\frac{m_{11}}{m_{22}}ur-\frac{d_{22}}{m_{22}}v+\frac{\tau_{E,v}}{m_{22}}\\ \dot{r}=\frac{m_{11}-m_{22}}{m_{33}}u\nu -\frac{d_{33}}{m_{33}}\;r+\frac{\left(F_{l}-F_{r}\right)\cdot B_{d}+\tau_{E,r}}{m_{33}} \end{array}\right.$$

In the dynamic positioning segment, since the direction and magnitude of thrust output from both sides of the propellers remain the same, $F_{l}=F_{r}$, and according to Equation (14), we can obtain $\tau_{r}=0$. We can reformulate the dynamic equation of the USV as follows:

(16) $$\left\{\begin{array}{c} \dot{x}=u\cos\psi -v\sin\psi \\ \dot{y}=u\sin\psi +v\cos\psi \\ \dot{\psi }=r\\ \dot{u}=\frac{m_{22}}{m_{11}}vr-\frac{d_{11}}{m_{11}}u+\frac{\left(F_{l}+F_{r}\right)\cdot \sin\varphi +\tau_{E,u}}{m_{11}}\\ \dot{v}=-\frac{m_{11}}{m_{22}}ur-\frac{d_{22}}{m_{22}}v+\frac{\left(F_{l}+F_{r}\right)\cdot \cos\varphi +\tau_{E,v}}{m_{22}}\\ \dot{r}=\frac{m_{11}-m_{22}}{m_{33}}u\nu -\frac{d_{33}}{m_{33}}\;r+\frac{\tau_{E,r}}{m_{33}} \end{array}\right.$$

### 3.4. Parameter Identification of Model

The parameter identification of the model of the target point tracking segment is carried out using the uniform linear motion experiment and the slewing motion experiment. Table 2 shows the basic parameters of the USV.

Based on the above parameters, it can be calculated that $m_{11}=34.64$, $m_{22}=60.02$, $m_{33}=10.24$. Next, the parameter identification of the coefficient matrix, *D*, is carried out. $d_{11}=\frac{\partial \tau_{u}}{\partial u},d_{22}=\frac{\partial \tau_{v}}{\partial v},d_{33}=\frac{\partial \tau_{r}}{\partial r}$.

In the control system designed in this paper, the control code *n* (0–700),which corresponds to the input control voltage of the propeller (0–4 V), is set so as to control the output power of the propeller, and the linear relationship between $n_{l}$ and the voltage, $V_{l}$, for a single propeller is:

(17) $$V_{l}=0.0057n_{l}$$

According to the propeller manual, the relationship between individual propeller thrust and controlled voltage is as follows:

(18) $$F_{l}=9.5V_{l}$$

So the combined force of the dual propellers in the forward direction of the USV is $F=\tau_{u}=9.5\left(V_{l}+V_{r}\right)$, and the moment $\tau_{r}=9.5B_{d}\left(V_{l}-V_{r}\right)$. So, given a control code, the thrust of the propeller can be obtained, and then the longitudinal or angular velocities produced by different thrusts can be measured. It is then possible to fit a relationship between thrust and velocity or angular velocity.

Substituting Equation (17) into Equation (18), the relationship between the combined force and the control code when advancing is obtained as follows:

(19) $$F=0.1083n_{l}$$

**Figure 5:**
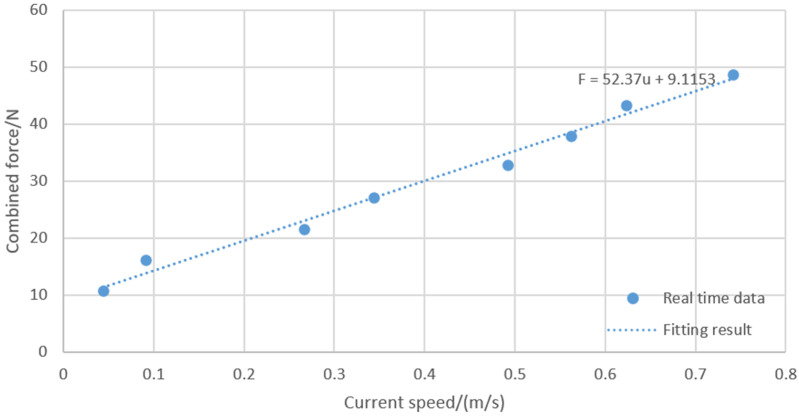
Linear relationship between velocity and combined force.

The experiment of uniform linear motion with different speeds was designed, and the real-time data were collected using the host computer to obtain the correspondence between the combined force and the current speed, and the experimental data are shown in Table 3. The fitting of the combined force and velocity is shown in Figure 5.

Linear relationship after fitting: F=52.37u+9.1153. Therefore, $d_{11}=\frac{\partial \tau_{u}}{\partial u}=\frac{\partial F}{\partial u}=52.37$.

Due to the experiment in a still-water lake, it is assumed that the lateral thrust of the USV in the tracking segment at the target point is negligible and the lateral velocity is 0. Therefore $d_{22}$ = 0, v=0.

Combining Equations (17) and (18), the relationship between control code and moment can be shown as follows:

(20) $$F_{r}=0.0227\left(n_{l}-n_{r}\right)$$

**Figure 6:**
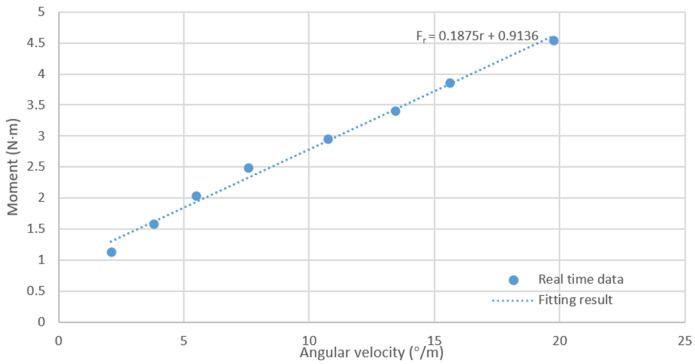
Linear relationship between angular velocity and moment.

Slewing experiments with different speeds were designed to obtain the relationship between moment and angular velocity, as shown in Table 4. The moments and angular velocities were fitted as shown in Figure 6. Therefore, $d_{33}=\frac{\partial \tau_{r}}{\partial r}=\frac{\partial F_{r}}{\partial r}=0.1875$.

In summary, by substituting the values of $d_{11},d_{22},d_{33},m_{11},m_{22},m_{33}$ into Equation (15) and ignoring the external perturbation, $\tau_{E}$, the mathematical model of USV motion can be obtained as follows:

(21) $$\left\{\begin{array}{c} \dot{x}=u\cos\psi -v\sin\psi \\ \dot{y}=u\sin\psi +v\cos\psi \\ \dot{\psi }=r\\ \dot{u}=-1.51u++0.029\left(F_{l}+F_{r}\right)\\ \dot{r}=-0.018r+0.041\left(F_{l}-F_{r}\right) \end{array}\right.$$

## 4. Target Point Tracking

During target point tracking, the bow angle of the USV initially needs to track the desired yaw angle and maintain it. In this segment, the output thrust of the propellers is always in the longitudinal direction of the hull, thereby satisfying the catamaran motion model described above. In this paper, a heading coordinate system is designed to calculate the desired yaw angle in real time by utilizing the position changes of the USV to always track the direction of the target point. The actual yaw angle is compared with the desired yaw angle to determine the working conditions of the propellers on both sides. Then, the angular cascade PID controller is utilized to adjust the rotational speed of the propellers on both sides to achieve the purpose of fast and accurate tracking of the desired yaw angle. Finally, a positional cascade PID controller is utilized to enable the USV to rapidly converge on the target position.

### 4.1. Establishment of the Heading Coordinate System

**Figure 7:**
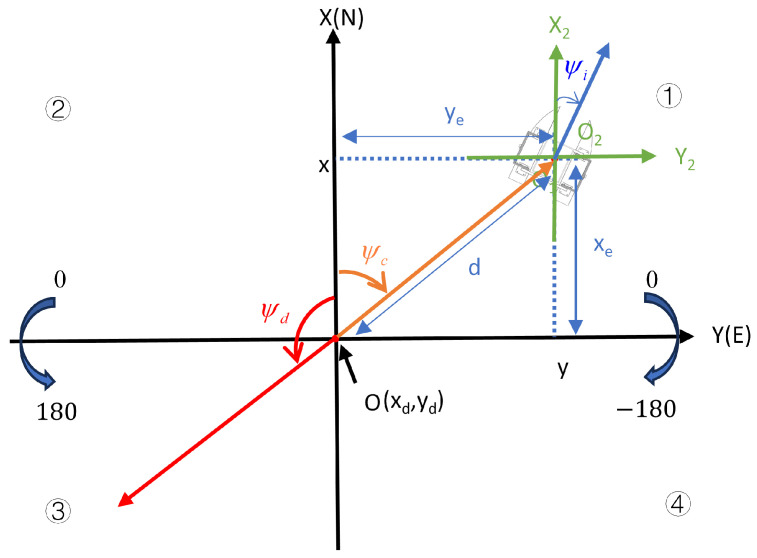
Schematic diagram of the heading coordinate system.

As shown in Figure 7, a two-dimensional heading coordinate system, OXY, is established. In this coordinate system, the direction of due north is taken as the positive direction of the *X*-axis and the direction of due east is taken as the positive direction of the *Y*-axis. The target point is set as the origin, *O*.

The latitude and longitude are transformed to planar coordinates through Mercator transformation. The coordinates of the target point are set as $\left(x_{d},y_{d}\right)$, The coordinates of the current position of the USV are set to (x,y). The longitudinal error, $x_{e}$, and lateral error, $y_{e}$, in the heading coordinate system are shown below:

(22) $$\left\{\begin{array}{c} x_{e}=x-x_{d}\\ y_{e}=y-y_{d} \end{array}\right.$$

Based on the positivity and negativity of $x_{e}$ and $y_{e}$, we specify in which quadrant the USV is located at the target point. Quadrant ➀➁➂➃ is as follows:

(23) $$➀\left\{\begin{array}{c} x_{e}>0\\ y_{e}>0 \end{array}\right.\;➁\left\{\begin{array}{c} x_{e}>0\\ y_{e}<0 \end{array}\right.\;➂\left\{\begin{array}{c} x_{e}<0\\ y_{e}<0 \end{array}\right.\;➃\left\{\begin{array}{c} x_{e}<0\\ y_{e}>0 \end{array}\right.$$

As shown in Figure 7, $\psi_{c}$ is the azimuthal yaw angle of the USV. After judging the quadrant where the USV is located, the angle values of $\psi_{c}$ in different quadrants are calculated, respectively, and the desired yaw angle, $\psi_{d}$, is finally obtained. The formula is as follows:

(24) $$\left\{\begin{array}{c} \psi_{c}=-90+\frac{\arctan(\frac{\left|x_{e}\right|}{\left|y_{e}\right|})\times 180}{\pi }\;\;➀\\ \psi_{c}=90-\frac{\arctan(\frac{\left|x_{e}\right|}{\left|y_{e}\right|})\times 180}{\pi }\;➁\\ \psi_{c}=90-\frac{\arctan(\frac{\left|x_{e}\right|}{\left|y_{e}\right|})\times 180}{\pi }\;➂\\ \psi_{c}=90+\frac{\arctan(\frac{\left|x_{e}\right|}{\left|y_{e}\right|})\times 180}{\pi }\;➃ \end{array}\right.$$

(25) $$\left\{\begin{array}{cc} \psi_{d}=\psi_{c}-180 & \;\psi_{c}>0\\ \psi_{d}=\psi_{c}+180 & \;\psi_{c}\leq 0 \end{array}\right.$$

A two-dimensional ship coordinate system, $O_{2}X_{2}Y_{2}$, is established, taking due north as the $X_{2}$-axis positive direction, due east as the $Y_{2}$-axis positive direction, and the center of the USV as the origin, $O_{2}$. In Figure 7, $\psi_{i}$ represents the actual yaw angle of the USV, which is the angle in the direction of the bow, and $\psi_{i}$ is obtained by IMU module after processing. By combined Figure 7, and Equations (24) and (25) to analyze, the value ranges of the azimuthal yaw angle, $\psi_{c}$, desired yaw angle, $\psi_{d}$, and actual yaw angle, $\psi_{i}$, are the same. All of them have a due north direction of 0, a clockwise rotation angle range of (0, −180), and a counterclockwise rotation angle range of [0, 180]. Therefore, as long as the values of $\psi_{i}$ and $\psi_{d}$ are equal, this indicates that the bow of the USV is pointing at the target point at this moment. In Figure 7, *d* is the distance between the USV and the target point, which is calculated as follows:

(26) $$d=\sqrt{x_{e}^{2}+y_{e}^{2}}$$

### 4.2. Design of Heading Tracking Controller

#### 4.2.1. Principles of Automatic Heading Control

The design idea of the heading tracking controller is to steer the USV by controlling the output moment of the propellers. By comparing the actual yaw angle, $\psi_{i}$, the desired yaw angle, $\psi_{d}$, and the azimuthal yaw angle, $\psi_{c}$, the operating status of the left propeller, $M_{L}$, and the right propeller, $M_{R}$, are determined. The operating state of the propeller determines whether the USV turns clockwise or counterclockwise, ultimately achieving the goal of turning the smallest possible angle to be able to track the desired heading of the USV.

**Figure 8:**
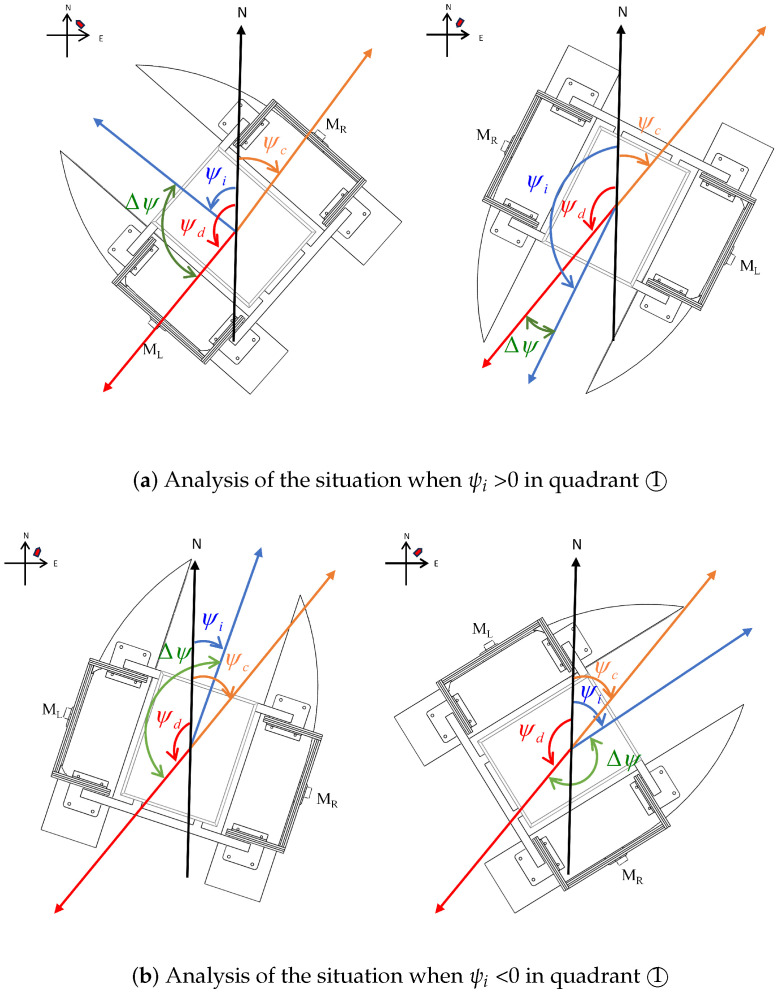
The principle of heading tracking.

As shown in Figure 8, it is assumed that the USV is in the first quadrant, and its actual position remains unchanged during the steering process. Additionally, the position of the target point is fixed. According to Equations (24) and (25), it can be analyzed that the values of $\psi_{c}$ and $\psi_{d}$ remain unchanged. The USV regulates the actual yaw angle through differential steering of the propellers, so only the value of $\psi_{i}$ changes. Since the value range of the angles in the heading coordinate system and the ship coordinate system are the same, the angular representation in the heading coordinate system can be discussed in the ship coordinate system. The two cases in Figure 8a are used as examples to analyze the operating state of the propellers when tracking the desired yaw angle.

As shown in Figure 8a, when the value of the actual yaw angle, $\psi_{i}$, is greater than 0. If $\psi_{i}$ is less than $\psi_{d}$, the right propeller, $M_{R}$, should rotate forward and the left propeller, $M_{L}$, should rotate backward. This is to push the USV to turn counterclockwise so that $\psi_{i}$ converges to $\psi_{d}$. If $\psi_{i}$ is greater than $\psi_{d}$, then the left propeller, $M_{L}$, rotates forward and the right propeller, $M_{R}$, rotates backward, pushing the USV clockwise so that $\psi_{i}$ converges to $\psi_{d}$.

As shown in Figure 8b, when the value of the actual yaw angle $\psi_{i}$ is less than 0. If $\psi_{i}$ is greater than $\psi_{c}$, the right propeller, $M_{R}$, should rotate forward and the left propeller, $M_{L}$, should rotate backward. This is to push the USV to turn counterclockwise so that $\psi_{i}$ converges to $\psi_{d}$. If $\psi_{i}$ is less than $\psi_{c}$, the left propeller, $M_{L}$, rotates forward and the right propeller, $M_{R}$, rotates backward, pushing the USV to turn clockwise so that $\psi_{i}$ converges to $\psi_{d}$.

**Figure 9:**
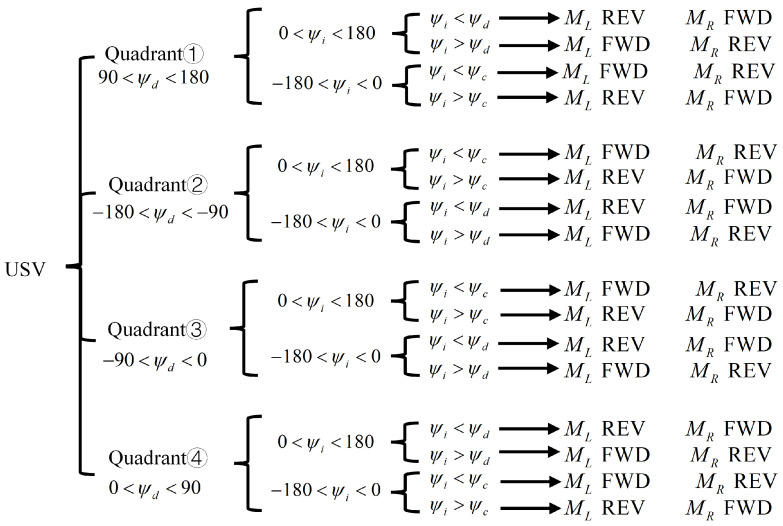
Propeller operations on both sides when tracking the desired yaw angle.

The control shown in Figure 9 only determines whether the propellers are operating in forward or reverse rotation and does not precisely control the rotational speed of the propellers. Therefore, a faster response bang-bang control is employed. In Figure 9, FWD indicates forward rotation and REV indicates reverse rotation. Regardless of the direction in which the USV is located with respect to the target point, the judgment principle shown in Figure 9 is used to quickly determine how the left propeller and the right propeller should work so that the USV can turn at the smallest possible angle to be able to track the desired yaw angle, thereby improving the response speed.

#### 4.2.2. Design of Heading Cascade PID Controllers

The USV not only needs to rapidly converge to the desired heading but also needs to accurately track the desired heading. When navigating in real water environments, the USV is bound to be affected by wind and waves. Thus, the USV must be able to complete target point tracking by adjusting its heading in real time when it is disturbed. In this paper, cascade PID controllers are employed to control the yaw angle and target point distance.

**Figure 10:**
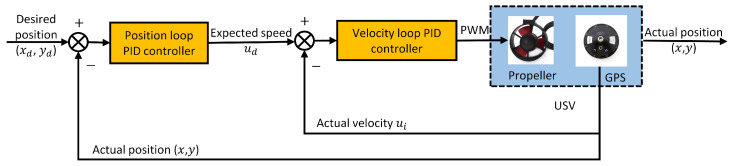
Angular cascade PID controller.

Figure 10 shows the process diagram of the angular cascade PID controller. In the process of tracking the target point, the USV first tracks the desired heading through steering. During the steering process, the USV measures the current actual yaw angle, $\psi_{i}$, in real time via the IMU module. At the same time, it calculates the desired yaw angle, $\psi_{d}$, based on the relative position of the USV and the target point. Finally, it obtains the yaw angle deviation, Δψ, which is calculated by the following formula:

(27) $$\Delta =\left|\psi_{d}-\psi_{i}\right|$$

(28) $$\left\{\begin{array}{c} \Delta \psi =\Delta \;\Delta \leq 180\\ \Delta \psi =360-\Delta \;\Delta >180 \end{array}\right.$$

This deviation value is fed into the PID controller of the outer loop, and the output equation of the outer loop PID controller is:

(29) $$r_{d}=K_{po1}\Delta \psi +K_{io1}\int \Delta \psi dt+K_{do1}\frac{d\Delta \psi }{dt}$$

The outer loop controller is calculated to output a desired angular velocity value, $r_{d}$. $K_{po1},K_{io1}$, and $K_{do1}$ are the proportional, integral, and differential coefficients, respectively, of the outer loop of the angular cascade PID controller.

The IMU module of the USV also measures the current actual angular velocity, $r_{i}$, and compares it with the output of the outer loop to obtain the yaw angular velocity deviation $\Delta r=r_{d}-r_{i}$. This deviation value is fed into the PID controller of the inner loop, which is given by the output equation of the inner loop PID controller:

(30) $$C_{a}=K_{pi1}\Delta r+K_{ii1}\int \Delta rdt+K_{di1}\frac{d\Delta r}{dt}$$

After calculation, the inner loop controller outputs the control signal, $C_{a}$. This control signal then generates the PWM to control the rotational speed of the propeller during steering. $K_{pi1},K_{ii1}$, and $K_{di1}$ are, respectively, the proportionality coefficients, integration coefficients, and differential coefficients of the inner loop of the angular cascade PID controller.

**Figure 11:**
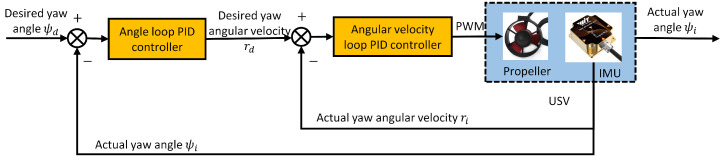
Positional cascade PID controller.

Figure 11 shows the process diagram of the positional cascade PID controller. Once the tracking of the yaw angle is achieved, the propellers on both sides maintain the same rotational speed to push the USV in the direction of the desired yaw angle and gradually converge to the target point. While moving forward, the USV measures the current actual position (x,y) in real time via the GPS module. The desired position $\left(x_{d},y_{d}\right)$ is set by the upper computer and sent to the ship-based controller through wireless communication. The actual distance between the USV and the target point can be obtained according to Equation (26). Let $e_{d}=d$, and input $e_{d}$ into the outer loop PID controller with the formula:

(31) $$u_{d}=K_{po2}e_{d}+K_{io2}\int e_{d}dt+K_{do2}\frac{de_{d}}{dt}$$

The outer loop is calculated to output the desired speed, $u_{d}$. $K_{po2},K_{io2},\;\mathrm{and}\;K_{do2}$ are the proportional, integral, and differential coefficients, respectively, of the outer loop of the positional cascade PID controller.

The GPS module simultaneously measures the actual velocity, $u_{i}$, and compares it with the output of the outer loop to obtain the velocity deviation $\Delta u=u_{d}-u_{i}.$ The velocity deviation, Δu, is input to the inner-loop PID controller, which is given by the formula:

(32) $$C_{d}=K_{pi2}\Delta u+K_{ii2}\int \Delta udt+K_{di2}\frac{d\Delta u}{dt}$$

The inner loop controller, after calculation, outputs the control signal $C_{d}$, which in turn yields the PWM to control the speed of the propeller as it advances. $K_{pi2},K_{ii2},\;\mathrm{and}\;K_{di2}$ are the proportional, integral, and differential coefficients of the inner loop of the positional cascade PID controller, respectively.

#### 4.2.3. Stability Analysis

Table 5 gives the parameter values of the cascade PID controllers and the values are obtained through actual experiments.

Combining Equations (18) and (21) yields the following equation:

(33) $$\left\{\begin{array}{c} \dot{u}=-1.51u+0.2755V_{1}\\ \dot{r}=-0.018r+0.3895V_{2} \end{array}\right.$$

where $V_{1}=V_{l}+V_{r},V_{2}=V_{l}-V_{r},V_{l},V_{r}$ represent the voltage of the left and right propellers, respectively. The transfer functions $G_{1}\left(S\right)$ and $G_{2}\left(S\right)$ are obtained according to Equation (33) as follows, respectively:

(34) $$\left\{\begin{array}{c} G_{1}\left(S\right)=\frac{0.2755}{s+1.51}\\ G_{2}\left(S\right)=\frac{0.3895}{s+0.018} \end{array}\right.$$

The transfer function, $G_{2}\left(S\right)$, is used as the controlled object of the angular cascade PID controller, so the open-loop transfer function of the angular cascade PID controller can be obtained as:

(35) $$G_{a}\left(S\right)=G_{a1}\left(S\right)G_{a2}\left(S\right)G_{2}\left(S\right)=\frac{0.642175s^{3}+1.900775s^{2}+0.027265s+0.00011685}{s^{2}\left(s+0.018\right)}$$

$G_{a1}\left(S\right),G_{a2}\left(S\right)$ are the transfer functions of the angular loop cascade PID controller and angular velocity loop cascade PID controller, respectively. The poles are found to be 0, 0, −0.018 according to the above equation. So the system is stable.

The transfer function, $G_{1}\left(S\right)$, is used as the controlled object of the positional cascade PID controller to obtain the open-loop transfer function of the system as:

(36) $$G_{p}\left(S\right)=G_{p1}\left(S\right)G_{p2}\left(S\right)G_{1}\left(S\right)=\frac{17.561875s^{3}\;+\;47.801375s^{2}+1.050625s+0.00391865}{s^{2}\left(s+1.51\right)}$$

$G_{p1},G_{p2}$ are the transfer functions of the position loop cascade PID controller and velocity loop cascade PID controller, respectively. According to the above equation, the poles are obtained as 0, 0, −1.51, so the system is stable.

## 5. Dynamic Positioning and Ship Experiment

The presence of various disturbing factors in the water environment will lead to the transverse drifting movement of the hull. The USV is differentiated from large vessels in that it does not have direct propellers in the transverse direction of the hull, and the actuator is prone to fall into thrust limitation. To address these issues, an adaptive adjustment mechanism is designed in this paper. This enables the propellers on both sides of the hull to no longer be limited to applying longitudinal thrust but to be able to flexibly distribute the thrust in all directions to compensate for the hull displacement caused by wind and wave currents. Finally, the whole process of the USV tracking the target point and remaining at the target point is verified through real ship experiments, proving the validity of the design.

### 5.1. Adaptive Adjustment Mechanism

#### 5.1.1. Hardware Architecture

**Figure 12:**
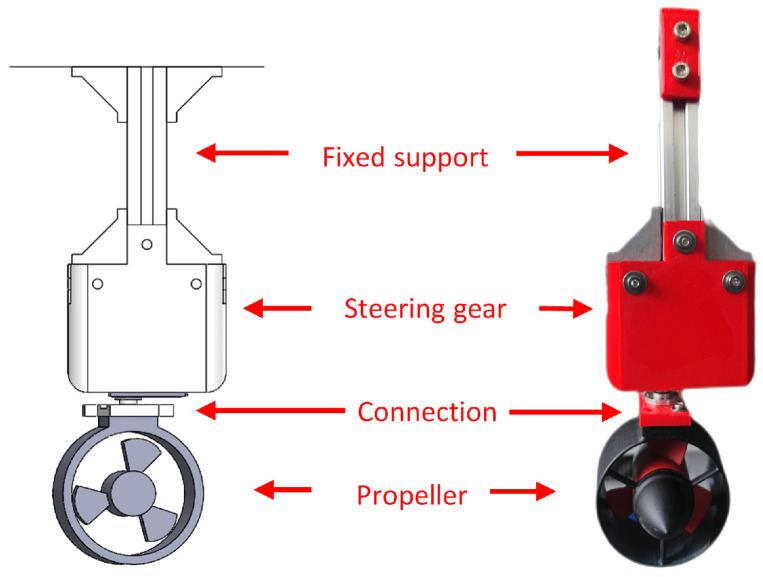
Hardware components of the adaptive adjustment mechanism.

As shown in Figure 12, the hardware component of the adaptive adjustment mechanism mainly comprises steering gears, propellers, connections, and fixed supports. The rotation of the steering gear drives the rotation of the propeller, thereby adjusting the direction of thrust and enabling the USV to output thrust in any direction. The aforementioned program provides hardware support to overcome the transverse drift motion.

#### 5.1.2. Principle of Adaptive Adjustment

**Figure 13:**
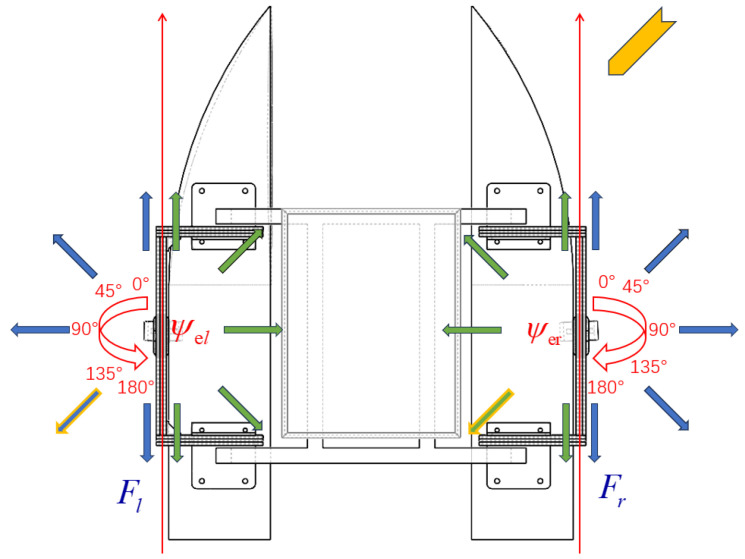
Schematic diagram of adaptive adjustment principle.

As shown in Figure 13, $\psi_{el}$ is the rudder angle of the left steering gear of the USV, and $\psi_{er}$ is the rudder angle of the right steering gear, both of which are oriented with the ship’s bow facing 0°. The angle ranges are shown in Figure 13. The blue arrows indicate the direction of thrust generated by the forward rotation of the propellers when the steering gears are rotated at different angles. The green arrows indicate the direction of thrust when the propellers are reversed. Although the steering gears rotate only 180°, the propellers can generate thrust in the opposite direction, allowing for a 360° thrust distribution. Assuming that the yellow arrow in the figure indicates the disturbance caused by wind and wave currents, in order to overcome the effects caused by this disturbance, the angle of the steering gears on both sides and the forward and reverse rotations of the propellers are controlled so that the thrust output of the USV is counteracted with the disturbance, which is represented as blue and green arrows with a yellow outer frame in Figure 13.

**Figure 14:**
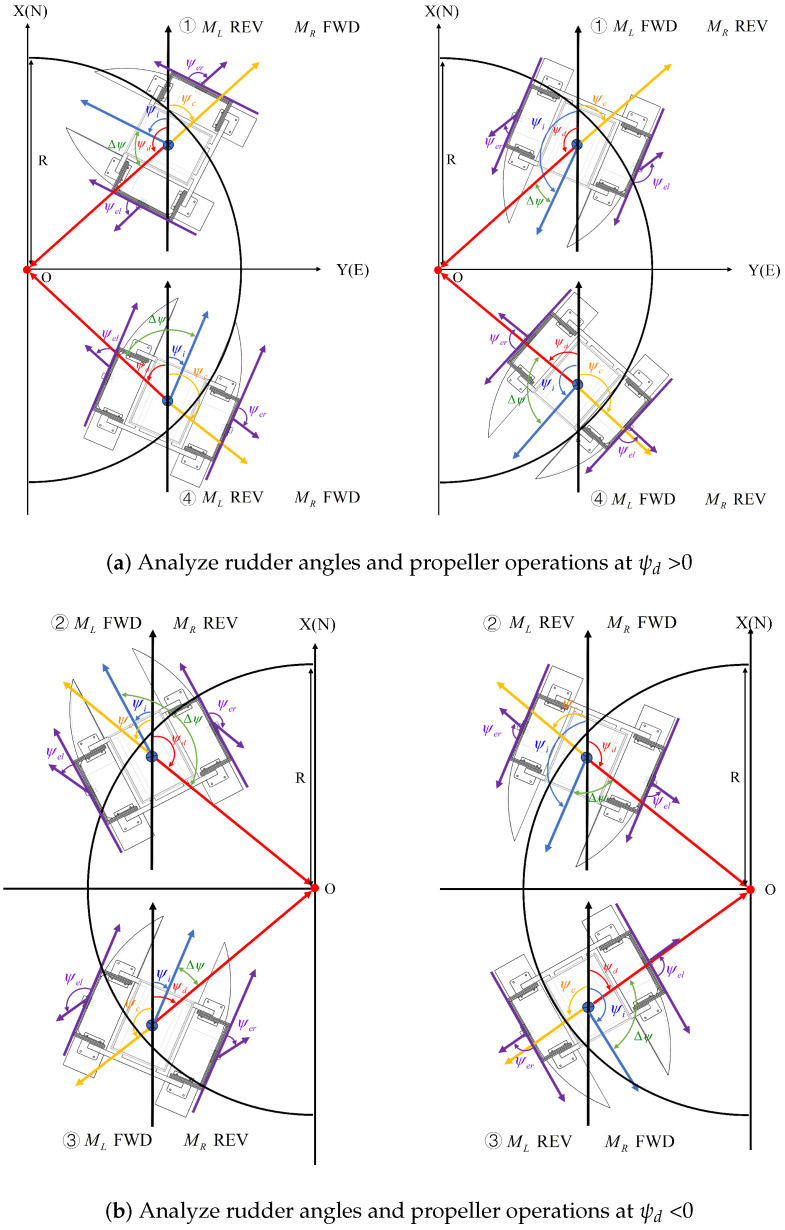
The angle of steering gears and the operation of propellers.

As shown in Figure 14, the rudder angles are analyzed using the heading coordinate system and the ship coordinate system in the event of transverse drift motion. If the USV is outside the circle, it is regarded as the target point tracking segment. At this time, the rudder angles on both sides are maintained at 180°. Regardless of the forward or reverse rotation of the propeller, the output thrust is only in the longitudinal direction. When the USV enters the circle, it is considered as a dynamic positioning segment. At this time, the steering gear starts to work to compensate for the transverse drift motion by changing the thrust direction.The following is an analysis of how the rudder angles are determined and how the propellers operate to generate thrust to compensate for the interference.

As shown in Figure 14a, the position of the USV is shifted within the circle due to interference. The desired yaw angle $\psi_{d}$ > 0, i.e., the USV is in quadrant ➀➃, at which point the relationship between $\psi_{i}$ and the values of $\psi_{d}$ and $\psi_{c}$ is compared. If the value of $\psi_{i}$ is $\left(\psi_{c},\psi_{d}\right)$, the left rudder angle, $\psi_{el}$, needs to be adjusted to Δψ, the left propeller, $M_{L}$, reversed, the right rudder angle, $\psi_{er}$, adjusted to 180−Δψ, and the right propeller, $M_{R}$, forwarded, in order to generate thrust toward the target point. If the value of $\psi_{i}$ is $\left[\psi_{d},180\right]\cup \left(-180,\psi_{c}\right]$, the left rudder angle, $\psi_{el}$, needs to be adjusted to 180−Δψ, the left propeller, $M_{L}$, forwarded, the right rudder angle, $\psi_{er}$, adjusted to Δψ, and the right propeller, $M_{R}$, reversed, in order to generate a thrust that tends to the target point.

Rudder angles $\psi_{el}$ and $\psi_{er}$ on both sides satisfy equation:

(37) $$\psi_{el}+\psi_{er}=180$$

As shown in Figure 14, the values of the rudder angles are related to the value of deviation angle Δψ between the actual yaw angle and the desired yaw angle. The angle of the steering gear on the side where the propeller is reversed is equal to the value of deviation angle Δψ, and the angle of the steering gear on the other side is 180−Δψ. Based on this logic, it is also possible to determine the rudder angles and propeller operations when the desired yaw angle $\psi_{d}$ < 0 in Figure 14b.

**Figure 15:**
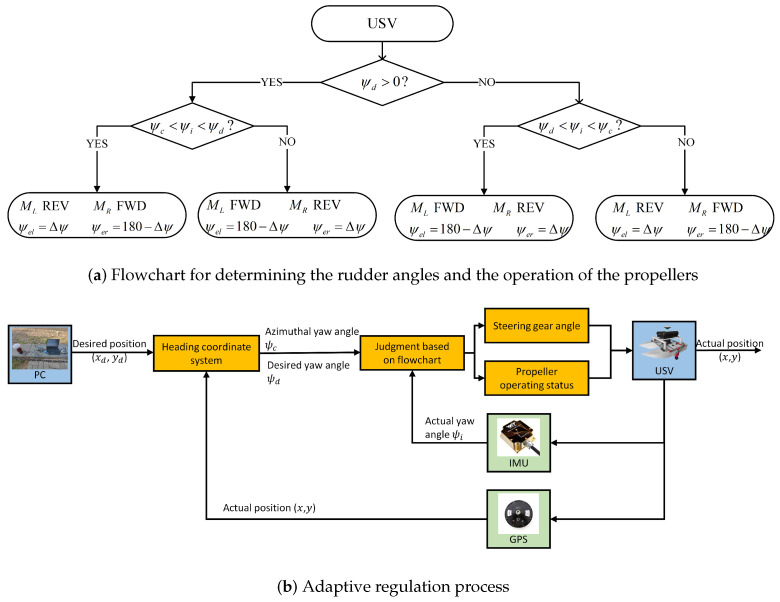
Overall design of the adaptive regulation process.

Figure 15 shows overall process for determining the operation of propellers and the angle of steering gears in different situations of the dynamic positioning segment. The deviation, Δψ, between the desired and actual yaw angle is changed in real time. The left rudder angle, $\psi_{el}$, and the right rudder angle, $\psi_{er}$, change in real time with the yaw angle deviation, Δψ, always outputting a thrust converging to the target point and constantly correcting the position of the USV. The above control is real time in nature. Accordingly, the position offset of the USV due to environmental perturbations can be minimized, and the accuracy of the positioning hold is improved.

### 5.2. Experiment

**Figure 16:**
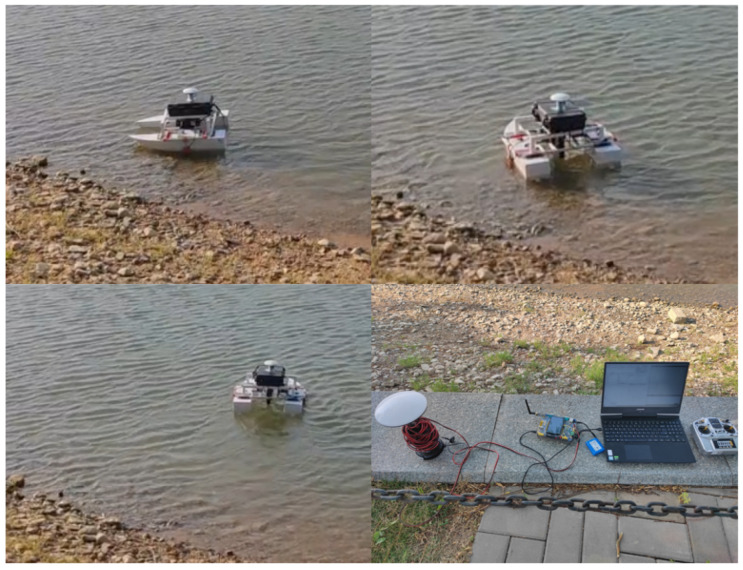
Schematic diagram of the experimental environment of the USV.

**Figure 17:**
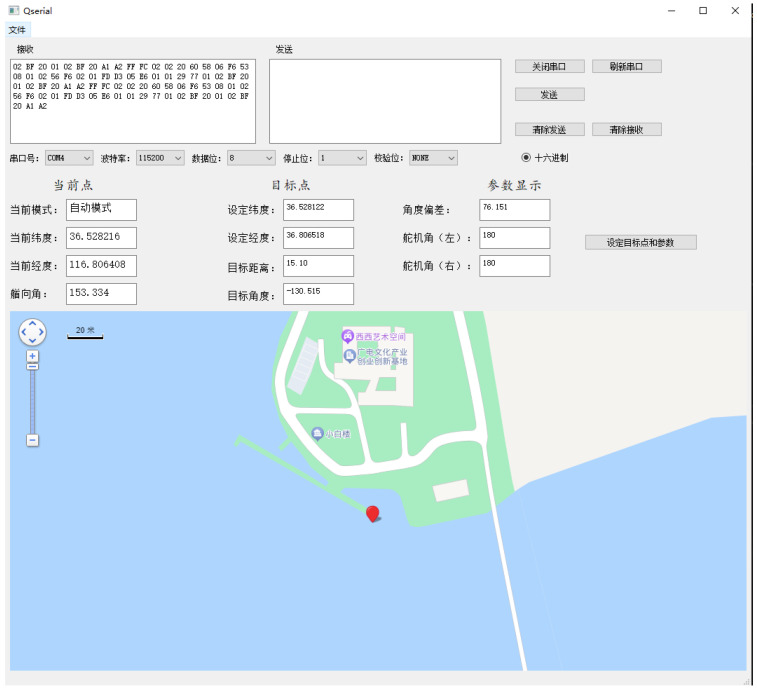
Upper computer information display.

In order to verify the application of the methodology used in this paper in real navigation control, navigation experiments were carried out on a lake within the Garden Expo Park in Changqing District, Jinan City. The data obtained from the USV were transmitted to the upper computer through the wireless module to display and create an Excel table to store the data. Figure 16 and Figure 17 show the experimental real picture and the schematic diagram of the upper computer.

After processing the redundant and anomalous data in the Excel sheet, the data in the sheet are plotted and analyzed by MATLAB.

In addition to analyzing the navigational data acquired by the USV as described above, it is also important to analyze the disturbances. Since it is difficult to observe the wind and waves directly, we use numerical simulation to estimate and measure the disturbance information. Since the experimental environment is a still-water lake, we focus on the effects of wind and current on the navigation experiments to give the basic model for calculating the wind interference force. The force and moment of the steady wind with direction $\alpha_{w}$ and velocity $U_{w}$ on the USV are expressed as:

(38) $$\left\{\begin{array}{c} X_{w}=-\frac{1}{2}\rho_{a}A_{T}\left(u_{rw}^{2}+v_{rw}^{2}\right)C_{wx}\left(\alpha_{w}\right)\\ Y_{w}=\frac{1}{2}\rho_{a}A_{L}\left(u_{rw}^{2}+v_{rw}^{2}\right)C_{wy}\left(\alpha_{w}\right)\\ N_{w}=\frac{1}{2}\rho_{a}A_{L}L\left(u_{rw}^{2}+v_{rw}^{2}\right)C_{wn}\left(\alpha_{w}\right) \end{array}\right.$$

where $u_{rw}$ and $v_{rw}$ are the speed of the ship after being affected by the wind, $\rho_{a}$ is the air density, $A_{T}$ and $A_{L}$ are the orthographic projection and measured projection areas above the USV’s waterline, respectively, and $C_{wx},C_{wy}$, and $C_{wn}$ are the wind coefficients in the *X* and *Y* directions and the wind moment coefficient around the *Z*-axis, respectively.

Assuming that the direction and velocity of the uniform current are $\alpha_{wa}$ and $U_{wa}$, the relative speed of the USV can be obtained as:

(39) $$\left\{\begin{array}{c} u_{rwa}=u+U_{wa}\cos\left(\alpha_{wa}-\psi \right)\\ v_{rwa}=v+U_{wa}\sin\left(\alpha_{wa}-\psi \right) \end{array}\right.$$

Table 6 gives the meteorological information and the basic parameters of the USV on the day of the experiment:

#### 5.2.1. Data Analysis of Target Point Tracking Segment

**Figure 18:**
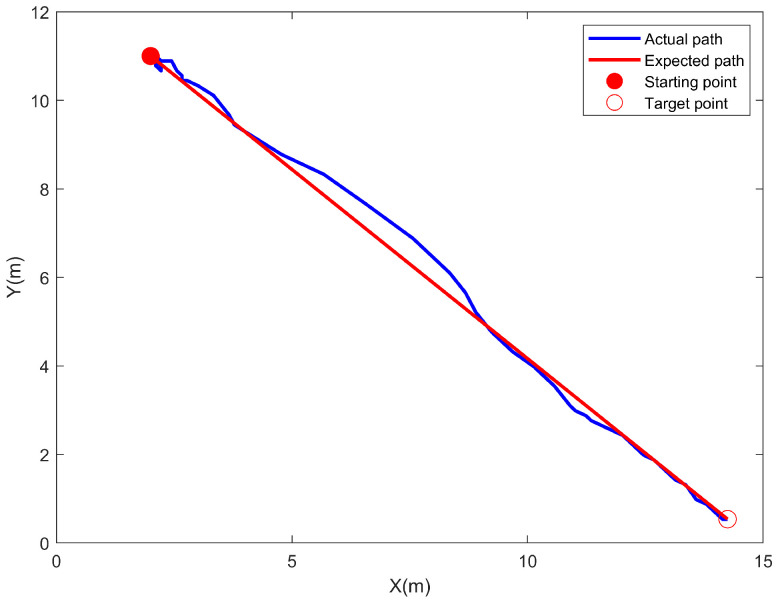
Schematic diagram of USV path for target point tracking segment.

As shown in Figure 18, the latitude and longitude are converted to planar coordinates for analysis after Mercator transformation. The horizontal coordinates are the transformed coordinates of the longitude information, and the vertical coordinates signify the transformed coordinates of the latitude information. The positive direction of the *Y*-axis is northward. In this paper, curved path following is not explored. Hence, the starting point and the goal point are connected by a straight line, which serves as the desired path. Initially, the USV tracks the desired yaw angle by differential steering, resulting in the USV moving irregularly around the starting point. After the steering is completed, the USV follows the desired path. However, there is uncertainty regarding the cause of the USV’s deviation in tracking the path since disturbance information like wind and wave currents is not directly measured. Nevertheless, after a significant deviation occurs, adjustments can be made to reduce the deviation from the desired path and fulfill the purpose of tracking to the desired path.

**Figure 19:**
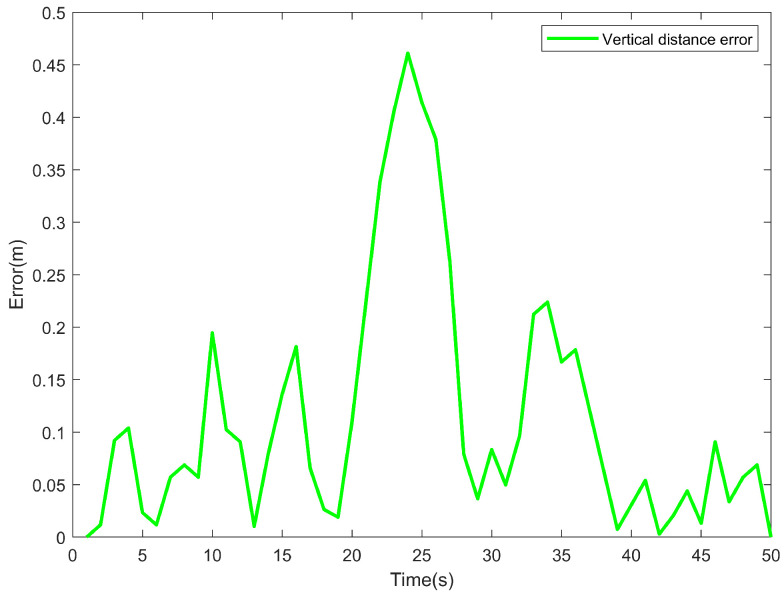
Vertical distance between the real-time position of the USV and the desired trajectory.

As shown in Figure 19, in order to visualize the effect of the USV tracking the desired path, a vertical distance figure between the actual trajectory points and the desired path is established. It can be observed from the figure that the vertical distance between the USV and the desired path is consistently less than 0.5 m. Since the width of the ship is 0.8 m and the GPS module is located in the center of the USV, the distance from the GPS module to either side of the USV is approximately 0.4 m. Thus, the deviation is within the acceptable error range of 0.5 m. Initially, the vertical distance deviation caused by adjusting the actual yaw angle was less than 0.2 m. Subsequently, due to interference factors, the deviation increased. However, after adjustment, the vertical distance was significantly reduced, indicating that the purpose of anti-interference was accomplished.

**Figure 20:**
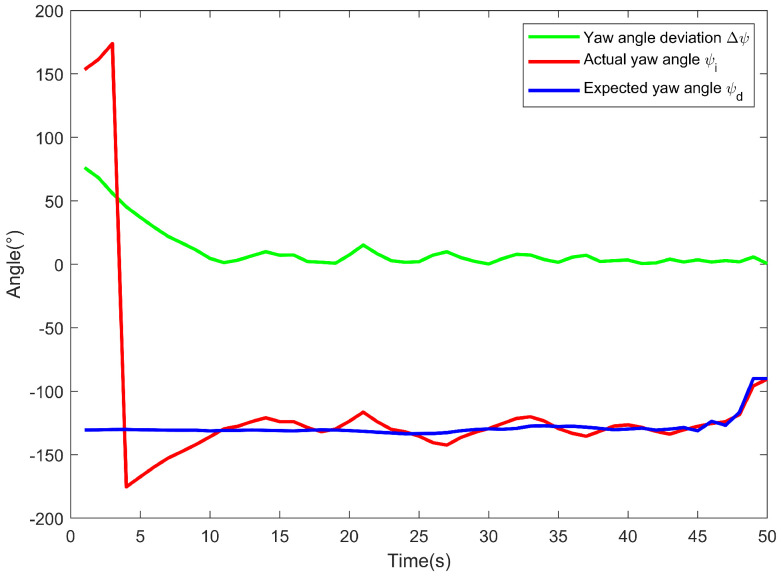
Comparison of desired yaw angle and actual yaw angle of USV.

As shown in Figure 20, the desired yaw angle, $\psi_{d}$, as an input to the angular cascade PID controller is based on the coordinates of the USV in conjunction with Equations (24) and (25). The actual yaw angle, $\psi_{i}$, as the output and feedback to the angular cascade PID controller is obtained directly from the IMU module. At the outset, the actual yaw angle of the USV was 153.334°, while the desired heading angle was −130.515°. In combination with the angle values of the coordinate system established in Section 4 and Figure 9, it is determined that the USV needs to turn counterclockwise in order to turn through the smallest angle to track the desired yaw angle, so the actual yaw angle first converges to 180° and then turns negative before converging to the desired yaw angle. As the motion progresses, the angular cascade PID controller regulates the yaw angle by adjusting the rotational speed of the propellers, and the yaw angle deviation gradually approaches a steady state, reflecting a excellent heading control effect and fulfilling the purpose of USV heading maintenance.

**Figure 21:**
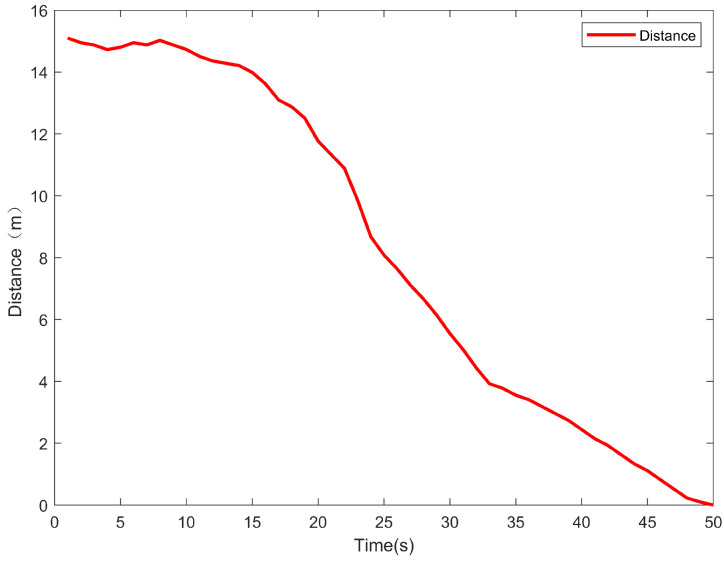
Distance between the USV and the target point in the target point tracking segment.

As shown in Figure 21, initially, the distance change is minimal due to the in-place differential steering of the USV for adjusting the yaw angle, which is consistent with the previous analysis. After adjusting the actual yaw angle to be consistent with the desired yaw angle, the distance from the target point decreases rapidly, signifying that the USV is moving swiftly toward the target point. Finally, the reduced speed for distance changes indicates that the positional cascade PID controller regulates the speed of the USV and prevents overshooting of the USV speed caused by excessive velocity.

#### 5.2.2. Data Analysis of Dynamic Positioning Segment

**Figure 22:**
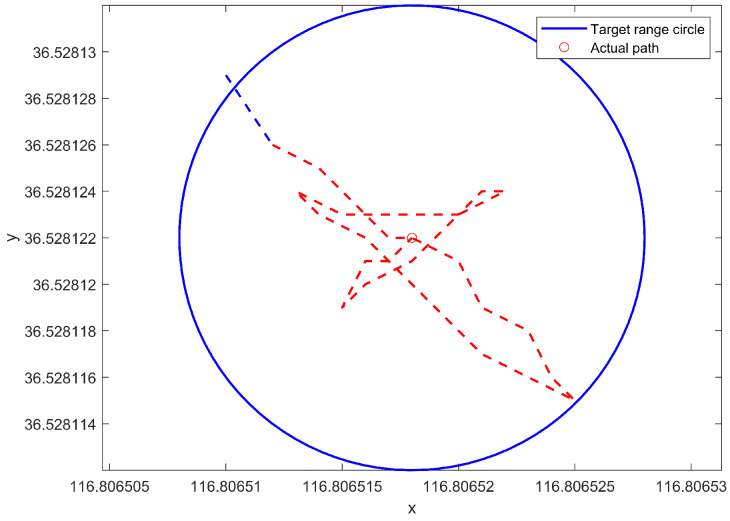
Schematic diagram of USV path for dynamic positioning segment.

As shown in Figure 22, latitude and longitude are converted to planar coordinates following Mercator transformation for analysis. The positive direction of the *Y*-axis is northward. Most of the paths shown in the figure are situated within a circle centered on the target point with a radius of 1 m. At this juncture, the steering gear begins to operate in conjunction with the propellers to adjust the actual position of the USV in the wind- and wave-disturbed environment. As can be observed from the figure, after the USV enters into the circle, it continuously adjusts near the target point. Since the GPS module is installed at the center of the USV, the path shown in the figure is the outcome of the transformation of the collected latitude and longitude information. Thus, the path showcases the adjustment process of the center of the USV. The length of the USV is 1 m and the width is 0.8 m. Although the center of the USV cannot be constantly maintained at the target point, the majority of the hull of the USV is within the circle and the distance from the target point is small in comparison with the size of the boat. Therefore, it essentially accomplishes the purpose of dynamic positioning.

**Figure 23:**
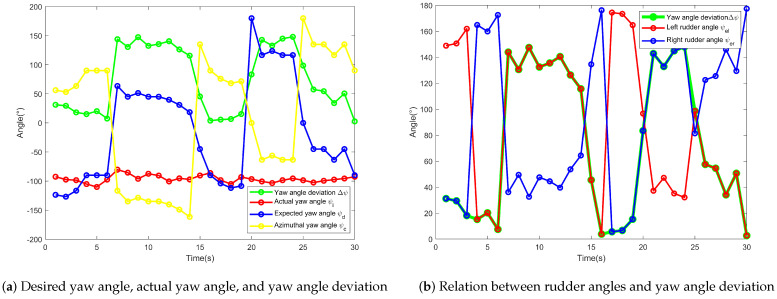
The analysis of rudder angles.

As shown in Figure 23, the relationship between yaw angles and rudder angles is illustrated. Figure 23a shows yaw angles for the dynamic positioning segment. According to Figure 22, it is evident that the USV enters different quadrants when adjusting its position, resulting in significant variations in the azimuthal yaw angle, $\psi_{c}$, and the desired yaw angle, $\psi_{d}$. In this segment, the USV controls rudder angles to distribute the thrust force. Since the magnitude and direction of the thrust forces are the same on both sides, based on Equation (14), it can be analyzed that the change in rudder angles of the USV does not generate a moment. Hence, it does not affect the actual yaw angle, and the variation in the actual yaw angle is insignificant. From the analysis of the flowchart presented in Figure 15, it can be noted that if $\psi_{d}$ > 0 and the value of $\psi_{i}$ is within the range $\left(\psi_{c},\psi_{d}\right)$, that is, when the red line is between the yellow line and the blue line, $\psi_{el}=\Delta \psi$ in Figure 23b, where the red line coincides with the green line. When the value of $\psi_{i}$ is within the range $\left[\psi_{d},180\right]\cup \left(-180,\psi_{c}\right]$, that is, when the red line is outside the yellow and blue lines, $\psi_{er}=\Delta \psi$ in Figure 23b, where the blue line coincides with the green line. Figure 23 also displays the values of the rudder angle for $\psi_{d}$ < 0, thereby verifying the feasibility of the adaptive regulation principle.

**Figure 24:**
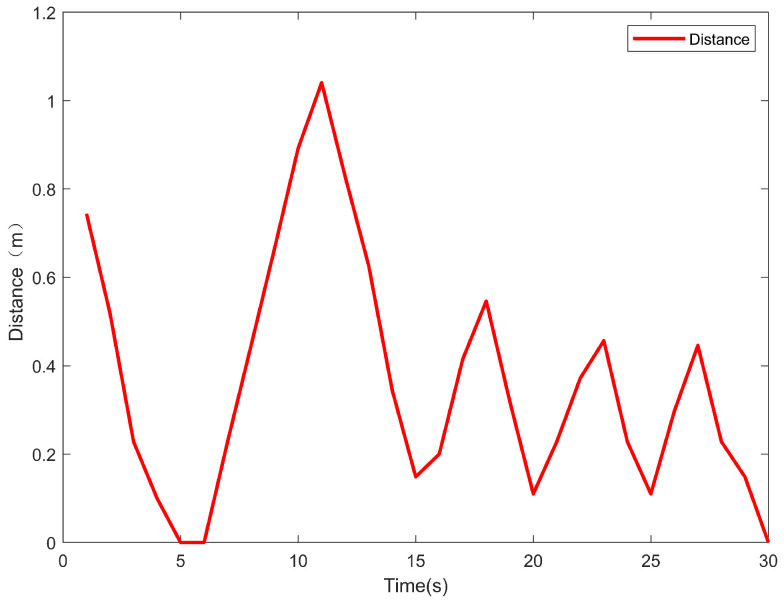
Distance between USV and target point in dynamic positioning segment.

As shown in Figure 24, in the dynamic positioning segment, the distance between the actual position of the USV and the target position fluctuates up and down, suggesting that the USV continuously adjusts its distance from the target point while remaining in the vicinity of the target point. Although the amount of interference such as wind and waves cannot be directly measured, it is inevitable that there are interference factors like wind and waves when experiments are carried out in real water environments. The distance gradually decreases from about 1 m to about 0.5 m. The distance between the USV and the target fluctuates up and down yet shows an overall downward trend. This indicates that the adaptive adjustment mechanism can reduce the deviation between the position of the USV and the target in the interference environment, thereby proving the effectiveness of the hardware and principle of the adaptive adjustment mechanism.

## 6. Conclusions

The objective of this paper is to investigate the motion control system for a small USV to rapidly track a target point and maintain it at the target point. To address the problem of tracking and maintaining the heading, the heading coordinate system and the ship coordinate system are established. Based on this, the value ranges of the angle in the coordinate system are specified. The desired yaw angle value is calculated in real time and compared with the actual yaw angle value to determine the working state of the propeller for carrying out differential steering. Additionally, it is combined with the angular cascade PID controller to regulate the speed of the propeller, enabling the USV to track and maintain the desired heading. The motion process is a straight-line motion from the starting point to the target point. Thus, path following is the tracking of the straight-line path. Thanks to the real-time updating of the control system, as soon as the position of the USV changes, the working state and rotational speed of the propeller are changed in real time, and real-time corrections are made to the motion path, achieving good straight-line path following results.

Upon the USV’s arrival at the target point, the existence of wind and waves makes it challenging for the USV to stay at the target point. To address this issue, an adaptive adjustment mechanism is designed to flexibly distribute the thrust force to counteract the force exerted by the wind and waves on the USV, thereby reducing the offset of the USV’s position. Finally, the effectiveness of the scheme presented in this paper is verified through real ship experiments. 

## Figures and Tables

**Table 1:** Representation of physical quantities in the coordinate system.

Serial Number	Description	Force (N)/ Moment (N·m)	Linear Velocity (m/s)/ Angular Velocity (rad/s)	Position/ Attitude Angle (°)
1	surge	X	*u*	*x*
2	sway	Y	*v*	*y*
3	heave	Z	*w*	*z*
4	roll	K	*p*	ϕ
5	pitch	M	*q*	θ
6	yaw	N	*r*	ψ

**Table 2:** The basic parameters of the USV.

Parameter	Value
Quantity m/kg	32.5
Length L/m	1.05
Height B/m	0.8
Draught depth at full load D/m	0.17
Distance from propeller to centerline $B_{d}$/m	0.42

**Table 3:** Velocity values under different uniform motions.

Control Code $n_{l}$	Control Code $n_{r}$	Combined Force F/N	Current Velocity/(m/s)
100	100	10.83	0.044
150	150	16.245	0.091
200	200	21.66	0.267
250	250	27.075	0.344
300	300	32.49	0.492
350	350	37.905	0.562
400	400	43.32	0.623
450	450	48.735	0.742

**Table 4:** Moment values and angular velocity at different speeds.

Control Code $n_{l}$	Control Code $n_{r}$	Moment $F_{r}$/N·m	Angular Velocity/(°/s)
150	100	1.135	2.085
170	100	1.589	3.791
190	100	2.043	5.494
210	100	2.497	7.558
230	100	2.951	10.748
250	100	3.405	13.429
270	100	3.859	15.623
300	100	4.54	19.742

**Table 5:** Parameter information of the controller.

Parameter Name	Value	Parameter Name	Value
$K_{po1}$	2.5	$K_{po2}$	23
$K_{io1}$	0.03	$K_{io2}$	0.11
$K_{do1}$	1.1	$K_{do2}$	8.5
$K_{pi1}$	1.5	$K_{pi2}$	7.5
$K_{ii1}$	0.01	$K_{ii2}$	0.13
$K_{di1}$	0	$K_{di2}$	0

**Table 6:** Basic parameter information.

Date of experiment	20 May 2024	Length of USV (m)	1.05
The direction of the wind	southwestern	Horizontal projected area (m^2^)	0.15
The speed of the wind (Kt)	7.5	Vertical projected area (m^2^)	0.072

## Data Availability

The data that support the findings of this study are available from the corresponding author upon reasonable request.
